# An efficient computer vision-based approach for acute lymphoblastic leukemia prediction

**DOI:** 10.3389/fncom.2022.1083649

**Published:** 2022-11-24

**Authors:** Ahmad Almadhor, Usman Sattar, Abdullah Al Hejaili, Uzma Ghulam Mohammad, Usman Tariq, Haithem Ben Chikha

**Affiliations:** ^1^Department of Computer Engineering and Networks, College of Computer and Information Sciences, Jouf University, Sakaka, Saudi Arabia; ^2^Department of Management Science, Beaconhouse National University, Lahore, Pakistan; ^3^Computer Science Department, Faculty of Computers & Information Technology, University of Tabuk, Tabuk, Saudi Arabia; ^4^Department of Computer Science and Software Engineering, International Islamic University, Islamabad, Pakistan; ^5^Department of Management Information Systems, Prince Sattam Bin Abdulaziz University, Al-Kharj, Saudi Arabia; ^6^Department of Computer Engineering and Networks, College of Computer and Information Sciences, Jouf University, Sakaka, Saudi Arabia

**Keywords:** leukemia prediction, acute lymphocytic leukemia, feature extraction, feature selection, machine learning, voting algorithm

## Abstract

Leukemia (blood cancer) diseases arise when the number of White blood cells (WBCs) is imbalanced in the human body. When the bone marrow produces many immature WBCs that kill healthy cells, acute lymphocytic leukemia (ALL) impacts people of all ages. Thus, timely predicting this disease can increase the chance of survival, and the patient can get his therapy early. Manual prediction is very expensive and time-consuming. Therefore, automated prediction techniques are essential. In this research, we propose an ensemble automated prediction approach that uses four machine learning algorithms K-Nearest Neighbor (KNN), Support Vector Machine (SVM), Random Forest (RF), and Naive Bayes (NB). The C-NMC leukemia dataset is used from the Kaggle repository to predict leukemia. Dataset is divided into two classes cancer and healthy cells. We perform data preprocessing steps, such as the first images being cropped using minimum and maximum points. Feature extraction is performed to extract the feature using pre-trained Convolutional Neural Network-based Deep Neural Network (DNN) architectures (VGG19, ResNet50, or ResNet101). Data scaling is performed by using the MinMaxScaler normalization technique. Analysis of Variance (ANOVA), Recursive Feature Elimination (RFE), and Random Forest (RF) as feature Selection techniques. Classification machine learning algorithms and ensemble voting are applied to selected features. Results reveal that SVM with 90.0% accuracy outperforms compared to other algorithms.

## 1. Introduction

The whole body receives crucial nutrients from the blood. Blood cells are divided into three primary types in the human body such as erythrocytes (red blood cells), leukocytes (white blood cells), and thrombocytes (platelets). Red blood cells (RBCs) ensure oxygen gets to all the body's cells. Platelets contribute to blood coagulation in the event of an accident. White blood cells (WBCs) defend against viruses and stop diseases in people. Only 1% of blood is made up of White blood cells because white blood cells are essential to the human immune system. Even small changes significantly impact (Sharif et al., 2020; Winter et al., 2021; Abir et al., 2022).

Diseases are indicated by an increase or decrease in the quantity of White Blood cells (WBCs) in blood plasma. There are five types of White Blood cells: lymphocyte, monocyte, neutrophil, eosinophil, and basophil. Any variation in the white blood cell count is cause for concern. Leukemia is the most frequently associated with a low White Blood cell (WBCs) count (Ghanem et al., 2019; Abir et al., 2022). It can be harmful and a factor in disease when our bodies have an unusually high quantity of WBC (Hegde et al., 2019).

Leukemia is a potentially deadly disease and is a type of cancer (Alfayez et al., 2021). It affects the blood and bone marrow due to the rapid growth of aberrant white blood cells. These aberrant WBCs cannot resist disease and affect the bone marrow's ability to make RBCs and platelets. There are two types of leukemia: acute and chronic. Acute leukemia grows faster and has more severe symptoms than chronic leukemia. The types of leukemia include lymphocytic and myelogenous. A type of white blood cell known as a lymphocyte contributes to the immune system. Lymphocytic leukemia describes excessive tumor growth in the cells in the bone marrow that become those lymphocytes. The hematopoietic cells that develop into RBCs, WBCs, and platelets grow abnormally in myelogenous leukemia. Four types of diseases classify leukemia, namely, Acute lymphocytic leukemia (ALL), Acute myelogenous leukemia (AML), Chronic lymphocytic leukemia (CLL), and Chronic myelogenous leukemia (CML).^1^

The most frequent type of childhood cancer is acute lymphoblastic leukemia (ALL) (Pan et al., 2017). The percentage of ALL cases involve healthy individuals, and only a small number of patients have genetic factors, including familial vulnerability or environmental factors. Chromosome abnormalities and genetic modifications related to lymphoid are its defining features (Godoy et al., 2020; Fujita et al., 2021). While ALL accounts for 80% of children's leukemia, it only accounts for 20% of adult leukemia. ALL is diagnosed using time-consuming and sophisticated procedures. The survival rate in developed nations has grown above 90% due to modern risk-adapted medicines and supportive care (Pan et al., 2017).

### 1.1. Motivation

In increasing healthcare problems, automated systems are essential (Kumar et al., 2022b). These systems can predict and classify acute lymphoblastic leukemia and are required to offer patients appropriate care and reduce its risks. The therapy and recovery of the patient depend heavily on the timely and precise detection of this type of cancer. Machine learning (ML) is a well-known scientific area based on artificial intelligence concepts that predict healthcare problems (Alsalem et al., 2018; Lalotra et al., 2021; Kumar et al., 2022a). Many researchers proposed techniques based on machine learning and deep learning algorithms for the early detection of acute lymphoblastic leukemia. Still, they are limited in providing better performance (Daqqa et al., 2017; Nazari et al., 2020) and did not consider feature extraction and selection techniques (Hossain et al., 2021). Considering these limitations, this research proposed an approach in which features are extracted using pre-trained CNN-based deep network architectures. The feature selection techniques are used to choose the relevant features from the large-size feature set acquired, and finally, classification is accomplished through machine learning techniques.

The main contributions performed for this proposed approach are given below:

- The pre-trained Convolutional Neural Network (CNN) based architectures VGG19, ResNet50, or ResNet101 are applied to enhance images by reducing noise and extracting the critical features.
- The feature selection techniques ANOVA, Recursive Feature Elimination (RFE), and Random Forest (RF) is applied to select the essential features that help to improve accuracy.
- The classification machine learning algorithms RF, SVM, KNN, NB, and Ensemble voting model, are applied to selected dataset features.
- The proposed model outperforms the classification of acute lymphocytic leukemia diseases and is also helpful for medical experts and patients by diagnosing acute lymphocytic leukemia early on time.

The rest of the paper is organized as follows. Section 2 presents the relevant literature. Section 3 provides the proposed approach. Section 4 presents the results and discussion. Finally, Section 5 concludes the research. Table 1 provides the list of abbreviations.

**Table 1:** List of abbreviation.

**Sr. no**	**Description**	**Abbreviation**
1	Acute lymphocytic leukemia	ALL
2	K-nearest neighbor	KNN
3	Support vector machine	SVM
4	Random forest	RF
5	Naive bayes	NB
6	Cancer dataset	C-NMC
7	Deep neural network	DNN
8	Recursive feature elimination	RFE
9	Convolutional neural network	CNN
10	Internet of medical things	IoMT
11	Principal component analysis	PCA
12	Machine learning	ML
13	Acute myelogenous leukemia	AML
14	Chronic lymphocytic leukemia	CLL
15	Chronic myelogenous leukemia	CML
16	Mathew's correlation coefficient	MCC
17	Receiver operating characteristic	ROC
18	Analysis of variance	ANOVA

## 2. Literature review

The techniques based on machine and deep learning algorithms used for the prediction of leukemia are explained in this section.

### 2.1. Machine learning techniques

Machine learning (ML) is gaining fast attention in various areas of cancer research (Abbas et al., 2021; Sacca et al., 2022; Safdar et al., 2022). ML is a promising tool for managing hematologic malignancies in the future due to its ability to interpret data from many diagnostic modalities, estimate mortality, and recommend treatment plans. ML-based techniques deal with different medical data and diseases. These ML approaches can be incorporated into numerous applications to guarantee rapid and accurate diagnosis, risk classification, and effective treatment (Eckardt et al., 2020). This article predicts leukemia using data mining techniques by examining the associations between blood characteristics and leukemia, gender, age, and patient health. Support Vector Machine (SVM), k-nearest neighbor (KNN), and decision tree (DT) are used for the prediction. 4,000 patient records are gathered from Gaza hospital. The experiment is performed, and accuracy and f1-measure evaluation metrics are used. Decision trees from all classifiers performed well with 77.30% accuracy (Daqqa et al., 2017).

The author proposed a supervised machine-learning approach for the early prediction of leukemia (Hossain et al., 2021). They primarily concentrate on typical symptoms and the possibility that a patient would eventually acquire leukemia. Typically, information about the features is provided at routine checkups. The used dataset is split into training and testing. Naive Bayes (NB), K-Nearest Neighbor (KNN), Random Forest (RF), Linear Regression (LR), Adaboost, etc., are used as machine learning algorithms to check out the performance of the model. Determined 17 parameters with a discussion with the doctor. Data is gathered from hospitals on various leukemia and non-leukemia patients. The highest result is achieved with a Decision Tree (DT) of 98%. In this article (Alsuwaidi et al., 2021), the author investigates novel genes demonstrating optimistic clinical and molecular signatures and uses a lymphoid cancer dataset. Lymphoblastic and lymphoid leukemia research comprising 1706 individuals and 2144 data are found in three different research. Only samples of B-lymphoblastic leukemia are chosen for additional examination. Using the cBioPortal tool, chromosomal alterations are evaluated to identify novel genomic loci to analyze clinical and molecular characteristics for the leukemia of lymphoid genesis. Homozygous deletions of the ADAM6 gene are found in 59.60% of the individuals analyzed and are linked to poor 10-year survival rates.

The author proposed an approach of supervised machine learning algorithms for the prediction of leukemia in the early stage by using symptoms. Dataset is gathered from the hospitals in Bangladesh. A survey on leukemia and non-leukemia patients is conducted with the help of a specialized doctor to gather 16 features for the datasets. Decision tree (DT) algorithms are supervised machine learning models. The Apriori algorithm is used to produce understandable principles for leukemia prediction. For comparison, many classifiers are applied to check the model's generalizability. Two feature selection and analysis steps are executed on the dataset to increase the model performance. The proposed model outperforms with 97.45% accuracy, 0.63 Mathew's Correlation Coefficient (MCC), and 0.783 of the area under the Receiver Operating Characteristic (ROC) curve (Hossain et al., 2022).

### 2.2. Deep learning techniques

The author of this research describes a model for detecting acute lymphocytic leukemia with separate depthwise convolutions (Clinton et al., 2020; Chatila et al., 2021). Diagnosing these tumor tissues as soon as possible is necessary to reduce the physical strain on the patient and the treatment difficulties that the disease brings (Sleiman et al., 2019; Aoun et al., 2020; Ali et al., 2022; Rizwan et al., 2022). Convolutional neural network (CNN) is tested using the transfer learning method. Xception model performance is evaluated on an extended microscopic blood smear image dataset. The model achieved 99% for the training dataset and 91% for the testing dataset. The 22283 gene leukemia microarray training dataset is taken from the Gene Expression Omnibus source. After designing and applying a deep neural network model to the data, classifiers cross-check the findings. Two preprocessing techniques are used for the data: normalization and principal component analysis (PCA). The findings demonstrate the PCA gene's capacity for independently segregating cancerous and healthy cells. The accuracy of the proposed model with three hidden layers and single-layer neural networks is 63.33 and 96.67, correspondingly (Nazari et al., 2020). Author in Genovese et al. (2021) proposed a methodology based on machine learning that uses an adaptive un-sharpening technique to improve images of blood samples. The approach normalizes the cell's radius using image processing algorithms, and deep learning estimates the focus reliability, adaptively enhances the sharpness of the images, and then does the classification. We tested the technique using an ALL publically available images dataset, taking into account several cutting-edge CNNs to carry out the classifying. The conclusions demonstrate the reliability of the proposed approach.

Author in de Oliveira and Dantas (2021) presents straightforward modifications to conventional neural network topologies to attain excellent performance in categorizing malignant leukocyte problems. Emulation, spinning, fading, shearing, and inserting salt-and-pepper noise are some of the changes used. The testing model used for the testing is VGG16, VGG19, and Xception. The model uses data augmentation to counteract the training and validation datasets. The model outperforms the evaluation metric f1-score 92.60%. The author proposed an Internet of Medical Things- (IoMT-) based approach to improve and nourish a fast and secure prediction of leukemia. The proposed system facilitates real-time leukemia screening, diagnosis, and treatment scenarios that save time and effort for both patients and medical experts and also address patients' issues in pandemics like COVID-19. Two convolutional neural networks (CNN) architectures, ResNet-34 and DenseNet-121, are used as a proposed model. The proposed model performed well-compared to other traditional algorithms. An experiment is performed on ASH and ALL-IDB images dataset accessible to the general public (Bibi et al., 2020).

In conclusion, many techniques are proposed based on machine learning and deep learning algorithms for predicting acute lymphoblastic leukemia (ALL). Still, they are limited in giving better performance and did not consider feature selection and extraction techniques. Considering these issues, this research proposed an efficient approach based on machine learning algorithms and ensemble algorithms to classify leukemia disease. Timely prediction of ALL diseases can reduce the mortality rate.

## 3. Proposed methodology

**Figure 1:**
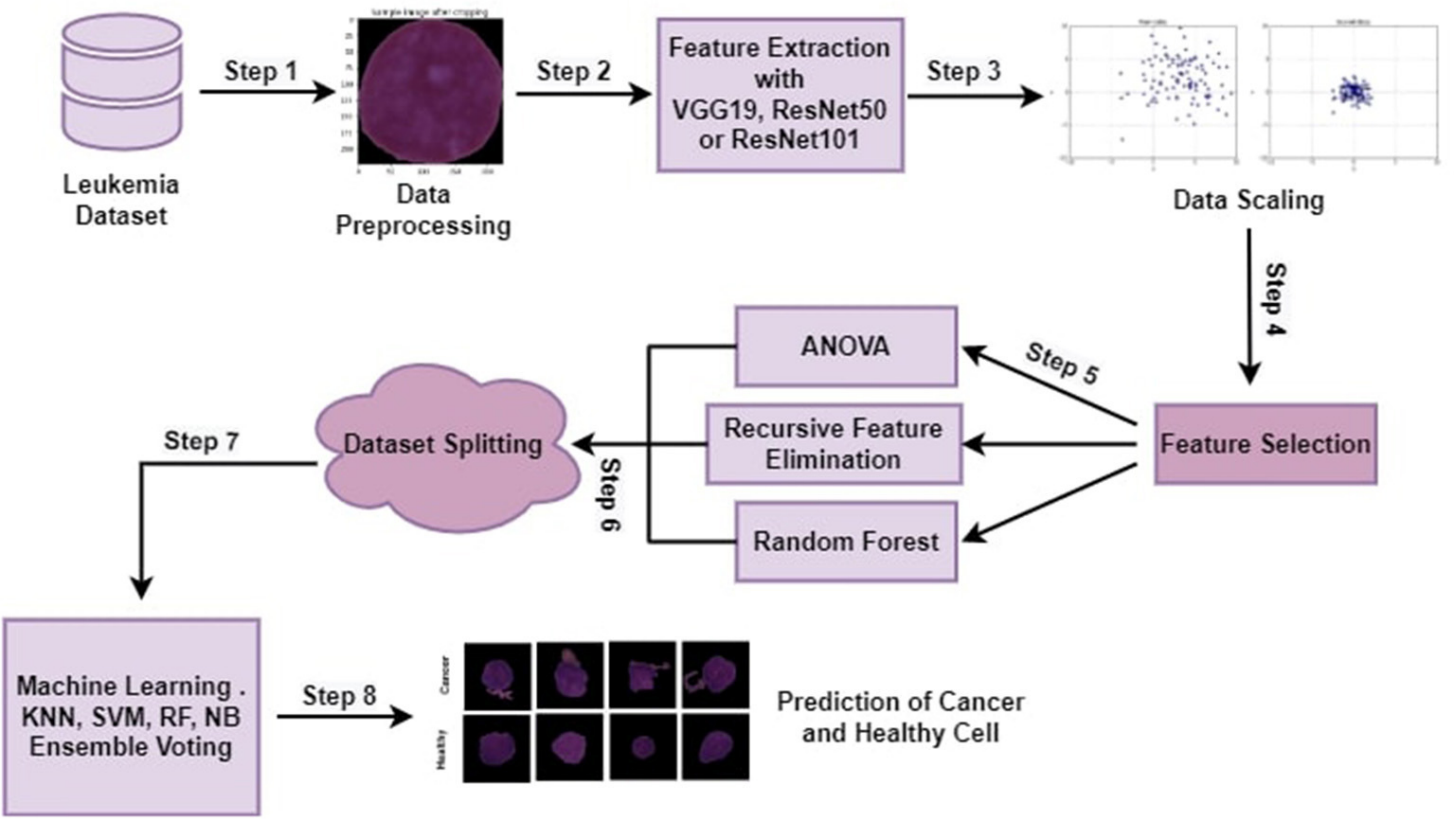
Proposed approach for acute lymphocytic leukemia prediction.

The proposed methodology with all steps is explained in this section in detail. Machine learning algorithms and ensemble algorithms are used to classify leukemia disease. Evaluation measurements such as accuracy, precision, recall, and f1-score are used to evaluate the proposed approach. This research experimented using a jupyter notebook on Anaconda. Python programming language is used for this experiment. This testing environment shows how programmers may create and evaluate machine learning (ML) models on a well-structured platform. Dynamic semantics are a feature of the high-level programming and interpreting language Python. The proposed approach has the following main steps cropping all the images by using minimum and maximum points, feature extraction through pre-trained CNN architectures (VGG16, ResNet50, or ResNet 101), Data scaling by applying MinMax normalization technique, feature selection through ANOVA, RFE, and RF. Selected features are utilized by machine learning algorithms and ensemble voting algorithms to classify leukemia. Figure 1 depicts the proposed approach steps.

### 3.1. Dataset

**Figure 2:**
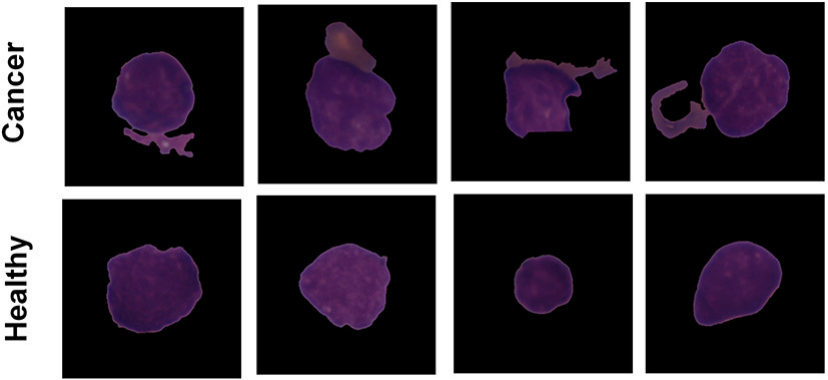
Leukemia images samples for two classes cancer and healthy cell.

The Proposed model performance is evaluated on the C-NMC leukemia dataset for leukemia cancer prediction. Dataset is available publicly and is collected from the Kaggle repository. The user can search and submit various datasets on the Kaggle repository, collaborate with other machine and deep learning publishers and data scientists, and develop and explore various models in various data science environments (Dahiwade et al., 2019). The leukemia dataset has 10,661 total images in the form of data. The dataset contains three leading folder names fold_0, fold_1, and fold_2. Every folder has two sub-folder with the name “all” and “hem.” The folder “all” has 0:7272 data equal to 1 data label, and “hem” has 7272:10661 data equal to 0 data labels. Figure 2 shows classes of the leukemia dataset.

### 3.2. Dataset preprocessing

**Figure 3:**
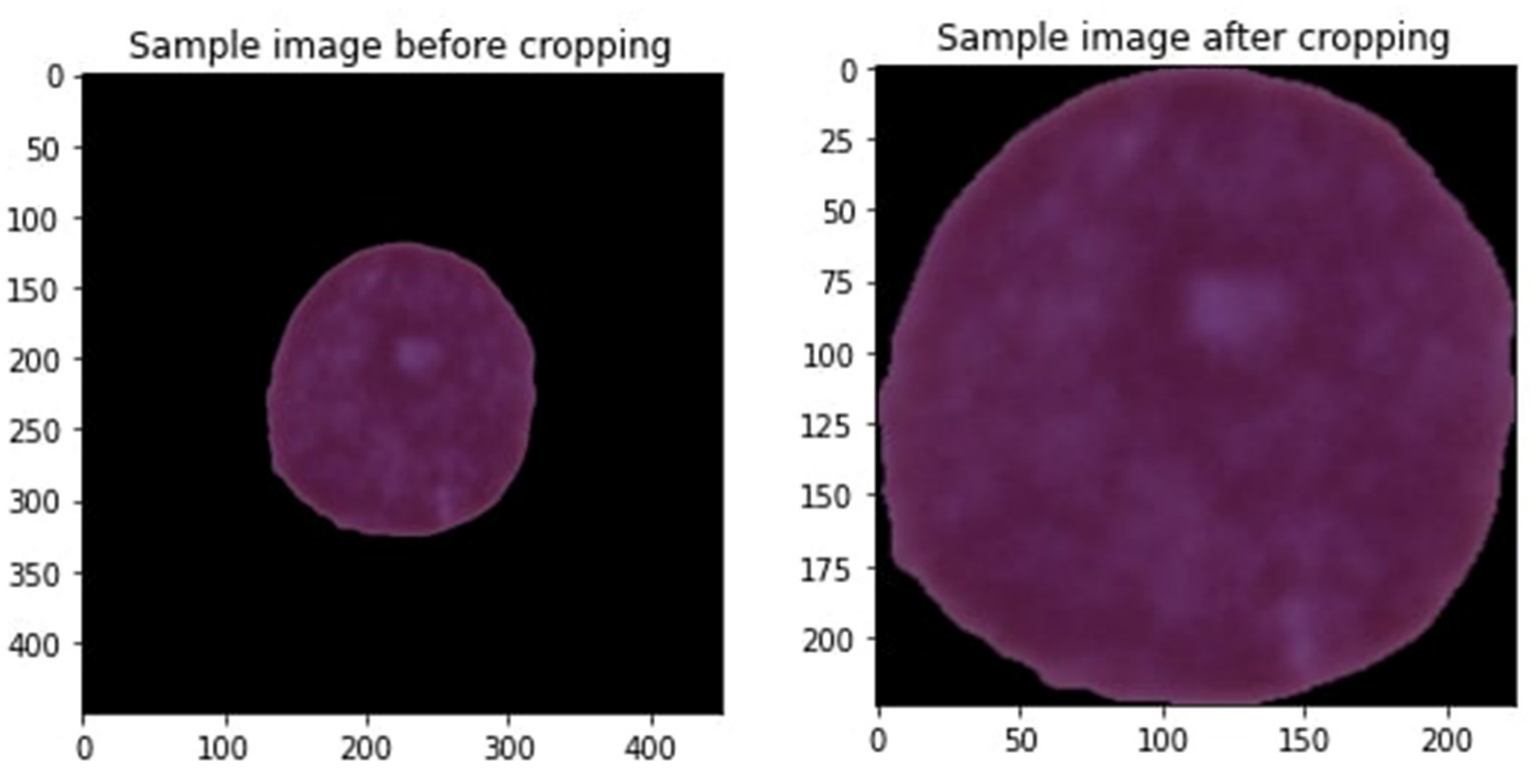
Cropping of sample image.

Data preprocessing is the preliminary step when designing any machine learning (ML) model for predicting diseases (Khan et al., 2019). Data preprocessing is executed to terminate the noisy data that are the reason for decreasing model performance. Every image of a leukemia dataset contains undesirable areas and empty spaces. Hence, cropping the images is essential to eliminate unnecessary space and use only vital information. We applied the extreme point calculation methodology for cropping images used in this study (Dahiwade et al., 2019). Figure 3 is the sample image we are cropping.

Some steps involve cropping the image, such as loading the leukemia image, transforming it into a grayscale, and then applying a threshold to the image to convert it to a binary image. Then, compute four separate minimum and maximum points (x,y) using the four most significant contours determined from the threshold images. At last, crop the image using the data gathered from minimum and maximum points and contours. All the images in the leukemia dataset have various sizes, heights, and widths, so it is essential to keep them in the same size with equal height and weight to improve the model's performance. After resizing all the images, the crop image size is 224 * 224. Range from 0 to 255 are used for all the images. Since there is greater noise around the edge, cropping the images is the best choice. Figure 3 is the sample image after cropping the image. Some more steps are involved in conducting data preprocessing that are explained below.

#### 3.2.1. Feature extraction

After cropping all images of the leukemia dataset, the next step is feature extraction. Feature extraction is the numerical representation of the used dataset. All the raw data is converted into useful information for applying machine learning (ML) models on the dataset. Model performance increased by applying this step compared to raw data for the ML model. This research used pre-trained Convolutional Neural network-based deep neural network architectures (VGG19, ResNet50, or ResNet101) to extract features from images. From all three CNN deep neural networks, we applied the ResNet50 architecture with a reshaped size (−1, 224, 224, 3). Several features are in 2,048 before applying feature selection techniques.

#### 3.2.2. Data scaling

It is essential to place dataset features into a scale equivalent when they are available on various scales. The standardization process involves rescaling the features to give them the properties of a typical normal distribution (Dahiwade et al., 2019). This research applied a MinMaxScaler normalization technique for scaling the leukemia dataset feature. Range defines for normalizing the features are 0–1.

#### 3.2.3. Feature selection method

Feature selection plays a significant role in the preprocessing stage of data analysis. Features must offer sufficient characteristics to categorize the data into classes or groups for further implication and decision-making in intelligent systems to achieve high accuracy. If only a few components are used, the results will not be sufficient. Performance becomes a problem if there are many features chosen. More essential features can be added to increase accuracy. In this research, we applied three feature selection techniques: ANOVA, Recursive Feature Elimination (RFE), and Random Forest (RF).

##### 3.2.3.1. ANOVA

Analysis of Variance (ANOVA) *F*-test statistics is a feature selection technique that removes insignificant features from the dataset (Olaolu et al., 2018). The parametric statistical hypothesis test determines whether the means from two or more data samples are from the same range. ANOVA is applied in this research as the first feature selection technique, and the k-value equals 500 (number of features).

##### 3.2.3.2. Recursive feature elimination

Recursive feature elimination (RFE), a feature selection technique, eliminates the weakest feature from a model until the required number of features is attained. RFE is applied in this research as the second feature selection technique. RFE aims to choose the best feature subset considering the learned model and classification accuracy (Jeon and Oh, 2020).

##### 3.2.3.3. Random forest

All the features in the dataset have different functions to classify. So some features that have less influence on the model's performance can be removed by applying the feature selection technique. Random forest algorithm has many benefits for selecting the features because it can quickly process the completion of high-dimensional data. Random feature selection can enhance classification performance (Li et al., 2020). RF is applied in this research as the third feature selection technique, and the value of the n-estimator is 200. After applying all techniques of feature selection, the number of features selected is 584. The leukemia dataset after preprocessing is split into 80% training and 20% testing for experimenting.

### 3.3. Machine learning algorithms

Machine learning algorithms such as K-nearest neighbors, Random forest, Support vector machine, Naive Bayes, and voting ensemble algorithm are applied as a proposed approach.

#### 3.3.1. K-nearest neighbors

K Nearest Neighbor (KNN) algorithm is a supervised machine learning algorithm applied to classification and regression problems. The knn algorithm has demonstrated impressive performance on data with many examples, such as near infinity, where its error rate roughly reaches the Bayes optimum under relatively moderate conditions. The performance of the KNN algorithm increases by choosing an optimal value of k (Zhang et al., 2017).

#### 3.3.2. Support vector machine

Support Vector Machine (SVM) algorithm is a supervised machine learning classifier that applies kernel trick procedure to determine the non-linear divisible problem by augmenting the data across a multi-resolution area. To find the best hyper-plane among the classes in the dataset, SVM classifiers extend the distance between the points closest to each class. The maximal hyper-plane gap widens the separation between the two classes (Pisner and Schnyer, 2020). This research used the SVM machine learning algorithm, and parameter values such as C = 100, gamma = 0.01, and kernel = “rbf” are used.

#### 3.3.3. Random forest

The random forest approach is based on developing many decision trees, each serving as a classifier. In random forests, a sub-dataset is created by sampling every tree in the forest from the given dataset. Each decision tree receives the sub-datasets, and each decision tree produces a conclusion. All decision trees vote to establish the final decision outcome (Li et al., 2020).

#### 3.3.4. Naive bayes

Naive Bayes is an essential learning algorithm that uses the Bayes rule and the fundamental presumption that, given the class, the attributes are conditionally independent (Webb et al., 2010). This independence assumption is frequently broken in practice, but naive Bayes produces competitive classification accuracy. It is also called a probabilistic classifier.

#### 3.3.5. Voting classifier

A voting algorithm is a machine learning classifier that develops several base models or classifiers. It makes predictions by combining their results. Voting for each classifier result can be integrated with the aggregating criteria. This research used the following base classifiers, RF, SVM, and NB, for making a voting classifier.

In Algorithm 1, this proposed approach work of all steps is described in detail. In this proposed approach, the input is the Leukemia dataset *L*_*d*_*s*, and *M*_*p*_ model performance is the output. Dataset is preprocessed by applying some steps such as minimum and maximum cropping techniques (*C*_*i*_) for cropping, an important feature is extracted using feature extraction (*f*_*extract*_), and CNN architectures VGG16, ResNet50, or ResNet101 are used. When the architecture of CNN is selected feature then reshapes (*Img*_*Reshape*_) the data. When the architecture of CNN is selected feature then reshapes (*Img*_*Reshape*_) the data. The next step is data scaling (*D*_*scaling*_) using the MinMax Normalization technique. The last step of preprocessing is feature selection *f*_*s*_. An important feature is selected by applying three techniques ANOVA, RFE, and RF. The leukemia dataset is split with 80% training data and 20% testing data. Simple machine learning classifier *Classifiers*_*ML*_ is applied to the training dataset (KNN, SVM, NB, RF). Then an ensemble voting (soft) model is applied, which contains three algorithms (RF, KNN, and SVM) with their hyper-parameters. The proposed model evaluated accuracy, precision, recall, and f1-score measurements.

**Algorithm 1:** Pseudo code of proposed approach.

1: *Input*: Leukemia dataset *L*_*d*_*s*
2: *Output*: Model Performance *M*_*p*_
3: *D*_*p*_ {data preprocessing}
4: *C*_*i*_ ← image[mnx:mxx,mny:mxy,:]
5: *f*_*extract*_ ← ResNet50
6: *Img*_*Reshape*_ ← VGG16 / ResNet50 / ResNet101
7: *D*_*scaling*_ ← MinMaxScalar
8: *f*_*s*_ ← Feature Selection
9: ANOVA
10: Recursive Feature Elimination
11: Random Forest
12: *x*_*train*_, *x*_*test*_, *y*_*train*_, *y*_*test*_ {Tain Test split}
13: *Classifiers*_*ML*_ ← KNN, SVM, NB, RF
14: *Ensemble*_*vot*_ ← Soft
15: *RF* ← n_estimators, max_depth
16: *KNN* ← n_neighbors
17: *SVM* ← C, kernel, gamma, probability
18: *E*_*m*_ ← accuracy, precision, recall, f1-score {Evaluation metrics}
19: Return ← Best Results

## 4. Result and discussion

This section explains the result of the proposed approach and used evaluation measurements in this research. The effectiveness of the proposed model for the prediction of ALL is discussed.

### 4.1. Evaluation measurements

Many evaluation measurements are utilized to identify the proposed model's classification results. This research used accuracy, precision, recall, and f1-score as evaluation measurements because many researchers used these measurements to evaluate the proposed methodology. Accuracy defines as calculating the ratio of true positive, false positive, true negative, and false negative. To find precision, compute the ratio of all positive values in the data. The recall is known as sensitivity, defined as computing the true positive by the true negative and false negative. F1-measure is calculated by taking the average of precision and recall measurements.

### 4.2. Experimental result

Table 2 demonstrates the overall performance of machine learning (ML) algorithms on the leukemia dataset. K-nearest Neighbor (KNN) algorithm achieved an accuracy of 83.4%, a precision of 84.1%, a recall of 93.3%, and an f1-score of 88.5%. The support Vector Machine (SVM) algorithm achieved an accuracy of 90.0%, a precision of 90.2%, a recall of 95.7%, and 92.9%. Random Forest (RF) achieved an accuracy of 82.6%, a precision of 82.1%, a recall of 95.3%, and an f1-score of 88.2%. Naive Bayes (NB) achieved an accuracy of 76.7%, a precision of 85.2% recall of 79.8%, and an f1-score of 82.4%.

**Table 2:** Performance of machine learning algorithm.

**ML**	**Accuracy (%)**	**Precision (%)**	**Recall (%)**	**F1-score (%)**
KNN	83.4	84.1	93.3	88.5
SVM	90.0	90.2	95.7	92.9
RF	82.6	82.1	95.3	88.2
NB	76.7	85.2	79.8	82.4
Voting	87.4	86.5	96.6	91.3

The voting classifier achieved an accuracy of 87.4%, a precision of 86.5%, a recall of 96.6%, and an f1-score of 91.3%. SVM performed better in accuracy, precision, and f1-score than other machine learning algorithms. The voting classifier performed well in terms of recall as compared to other machine learning algorithms.

**Figure 4:**
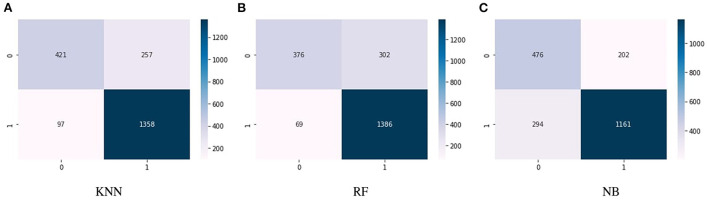
Confusion matrix of the classifiers. **(A)** KNN. **(B)** RF. **(C)** NB.

**Figure 5:**
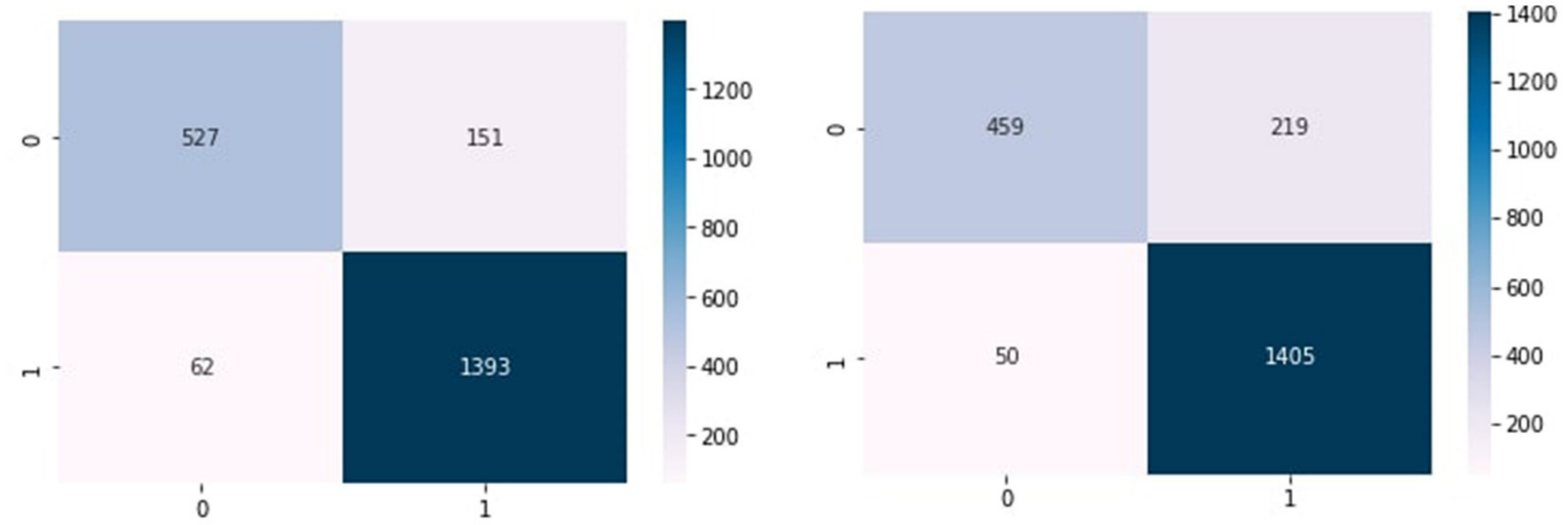
Confusion matrix of SVM and voting classifier.

Figures 4A–C show the confusion matrix of KNN, RF, and NB algorithms. These graphs express machine learning working. The proposed model performance increases if true positive and negative values are higher than false positive and negative values. Figure 5 presents the confusion matrix of the SVM (left side) and voting classifier (right side).

**Figure 6:**
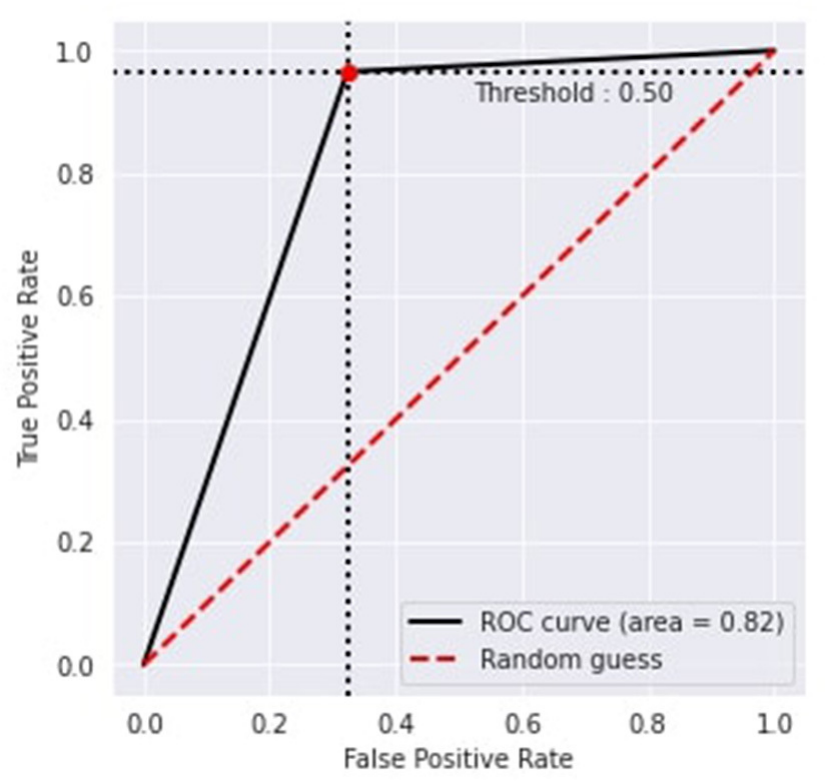
Recursive operating characteristic.

Figure 6 represents the proposed approach's recursive operating characteristics (ROC). The area of the ROC curve is 0.82% with a threshold value of 0.50, which makes the model better than the traditional model.

## 5. Conclusion

This research proposed an approach for the prediction of Acute Leukemia (ALL) based on machine learning algorithms (RF, SVM, KNN, NB) and ensemble voting classifier with a pre-defined CNN architecture (VGG16, ResNet50, or ResNet101) based feature extraction techniques. SVM performed well-compared to other algorithms with 90.0% accuracy for predicting ALL diseases. The voting classifier has 87.4% accuracy, which is less than the SVM algorithm but better than other algorithms. The result demonstrates that the proposed approach delivered more precise results. The C-NMC leukemia dataset is used from a kaggle repository. Following data preprocessing steps are performed, such as, every image is cropped by using minimum and maximum points techniques. The feature is extracted by applying ResNet50 architecture, and MinMax Normalization is used for data scaling. Three techniques: ANOVA, Recursive Feature Elimination (RFE), and Random Forest (RF), are applied for feature selection. Then preprocessed data are utilized as input for the ML algorithms to predict ALL. Thus, the proposed approach provides practitioners with a reasonable means of determining whether or not a patient has ALL. In the future, model generalizability will be checked by applying multiple datasets.

## Data availability statement

The original contributions presented in the study are included in the article/supplementary material, further inquiries can be directed to the corresponding author/s.

## Author contributions

All authors listed have made a substantial, direct, and intellectual contribution to the work and approved it for publication.

## Funding

This work was funded by the Deanship of Scientific Research at Jouf University under Grant No. (DSR-2021-02-0354).

## Conflict of interest

The authors declare that the research was conducted in the absence of any commercial or financial relationships that could be construed as a potential conflict of interest.

## Publisher's note

All claims expressed in this article are solely those of the authors and do not necessarily represent those of their affiliated organizations, or those of the publisher, the editors and the reviewers. Any product that may be evaluated in this article, or claim that may be made by its manufacturer, is not guaranteed or endorsed by the publisher.

## Footnotes

^1^ https://www.hematology.org/education/patients/blood-cancers/leukemia
